# Diet-derived male sex pheromone compounds affect female choice in a noctuid moth

**DOI:** 10.1038/s41598-023-47041-8

**Published:** 2023-11-13

**Authors:** Naomi L. Zweerus, Michiel van Wijk, Coby Schal, Astrid T. Groot

**Affiliations:** 1 Institute for Biodiversity and Ecosystem Dynamics, University of Amsterdam, Amsterdam, The Netherlands; 2 Department of Entomology and Plant Pathology, North Carolina State University, Raleigh, USA

**Keywords:** Evolution, Sexual selection

## Abstract

Sexual signals often function in species recognition and may also guide mate choice within a species. In noctuid moths, both males and females may exercise mate choice. Females of the tobacco budworm *Chloridea virescens* prefer to mate with larger males, but the signal(s) underlying female choice remain unknown. Male hairpencil volatiles are emitted during close range courtship displays. However, previously identified male hairpencil volatiles, namely acetate esters, aldehydes, alcohols, and fatty acids, are not associated with female choice. Recently, two new hairpencil compounds were identified that elicit strong electrophysiological responses in female antennae: methyl salicylate (MeSA) and δ-decalactone. In this study, we investigated the effect of larval diet and adult feeding on MeSA and δ-decalactone content in hairpencils and determined whether these compounds are involved in female choice. We found that larval diet affected MeSA content in hairpencils, but not δ-decalactone. Conversely, adult feeding affected the level of δ-decalactone, but not MeSA: sugar-water feeding increased δ-decalactone content compared to plain water. In two-choice assays, females mated more with males that had higher amounts of δ-decalactone, and less with males with higher amounts of MeSA.

## Introduction

Sexual signals used in mate choice are often subject to directional selection^1–3^ whereas sexual signals for species recognition are under stabilizing selection^4–7^. However, the same sexual signals may serve both functions; signals involved in species recognition may also make the signaller more attractive to the opposite sex, which may result in sexual selection on the whole or parts of these signals^2^. Hence, choice between a conspecific and a heterospecific mate or between two conspecific mates that differ in signal quality may represent a continuum^8–10^.

Moth sex pheromones, which are usually composed of multi-component blends, represent an excellent system to study how different functions of the pheromone and the associated selection pressures shape the evolution of sexual communication. In moths, females emit a species-specific sex pheromone to attract conspecific males from a distance^11^. Upon arrival, males often release a short-range chemical signal from structures, called hairpencils, that are extruded or everted from the abdomen^12,13^. Although the biological role of these male-specific volatiles has not been studied extensively, they have been implicated in a variety of processes related to mating^14–16^. For example, the male-specific volatiles may minimize cross-species attraction, as does the female sex pheromone^16–18^. Hairpencil compounds have been reported to have an aphrodisiacal effect on conspecific females, making them more quiescent and more likely to accept males^14,19^. Similar conclusions have been drawn from studies where hairpencils were ablated and mating behavior was (partially) rescued with the addition of hairpencil extract^16,20^ or where the production of hairpencil compounds was inhibited using RNAi^21^. Hairpencil volatiles have also been shown to play an anti-aphrodisiacal role for conspecific males and function as a chemical mate-guarding pheromone when applied to the female during mating^22^. Finally, the male hairpencil volatiles could inform females about male quality^14^. This aspect was best studied in the bella moth *Utetheisa ornatrix*, where females select larger males based on the concentration of hydroxydanaidal, a hairpencil compound that is derived from a plant-produced pyrrolizidine alkaloid^23,24^. Larger males provide (a) more defensive pyrrolizidine alkaloids in their spermatophores that the females use to protect their eggs and (b) indirect benefits through the acquisition of alleles associated with large body size^23,25^.

A male sex pheromone has been identified from the hairpencils of the tobacco budworm, *Chloridea* (formerly *Heliothis*) *virescens*^26^. Conspecific, but not heterospecific, hairpencil extract induced mate acceptance behavior in females, suggesting a role in species recognition^16^. A recent study revealed that female mate choice also occurs in this species^27^. However, while *C. virescens* females prefer to mate with relatively larger males, none of the acetate esters, aldehydes, alcohols, or fatty acid hairpencil compounds described by Teal and Tumlinson^26^ predicted female choice in *C. virescens*^27,28^. Interestingly, in re-examining the chemical composition of the male hairpencil pheromone in this species, two novel compounds were discovered that are perceived by the female antennae^29^: methyl salicylate (MeSA) and δ-decalactone. MeSA was shown to be sequestered by larval males from soybean leaves and from MeSA-supplemented diet and by adult males from MeSA-supplemented sugar water^29^. Further investigations of MeSA showed that this compound promotes mating success and may serve as a close-range aphrodisiac pheromone compound in *C. virescens*^29^. The function of δ-decalactone has not yet been determined.

In this study, we evaluated if MeSA and δ-decalactone are involved in female choice in *C. virescens*. Since both compounds can be sequestered from the diet or biosynthesized from dietary nutrients, we expected larger males to have acquired more resources and thus, to contain more MeSA and δ-decalactone. We then expected that females would prefer to mate with males with higher amounts of both compounds in their hairpencils. We first quantified the levels of known male pheromone compounds in adult males that were reared as larvae on diet with either full or reduced nutritional value, as well as in males that were given sugar water or plain water as adults. Since female choice is related to male size^27^, we then assessed whether and how male pupal mass was correlated to the abundance of all male pheromone compounds, including MeSA and δ-decalactone. Finally, to assess if females use these compounds as chemical signals for mate choice, we reanalyzed the data of female choice described in Zweerus et al.^27^, including MeSA and δ-decalactone.

## Methods

### Study organism

Field-collected *Chloridea* (formerly *Heliothis*) *virescens* moths have been reared at North Carolina State University since 1989^30^. The lab strain (YDK strain) was later also reared at the Max Planck Institute for Chemical Ecology, Jena since 2007, and at the Institute for Biodiversity and Ecosystem and Dynamics (IBED), University of Amsterdam since 2011. At all locations, the moths were reared in environmental chambers at approximately 60% relative humidity and 25 ± 1 °C, with a 14 h light (photophase): 10 h dark (scotophase) photoperiod. Larvae were reared on artificial pinto bean diet^31^ in individual plastic cups (37 ml, Solo, Lake Forest, Illinois). Pupae were checked regularly for eclosion and newly emerged adults were fed 10% sucrose solution provided through a soaked cotton dental wick. All experiments in this study were conducted with 2–3-day old individuals and under the same environmental conditions as the rearing conditions.

### Male hairpencil pheromone: extraction and analyses

To analyze the male hairpencil pheromone composition of individual males, we extracted the male hairpencils, following the protocol of Hosseini et al.^22^ as described in Zweerus et al.^27^. In short, we removed the hairpencils from the male at 2–3 days of age during the scotophase (dark period) and extracted the hairpencils in hexane with 200 ng of pentadecane (C15) as an internal standard for 30 min. We then concentrated the pheromone extract to 2–3 µl under a gentle flow of nitrogen, after which the complete sample was injected into the gas-chromatograph (GC), following the GC procedure described in Groot et al.^32^. To be able to align the retention times of the peaks of relevant compounds for integration, we also ran a synthetic multiple-component blend at regular intervals (every ~ 30 extraction runs). The synthetic blend included the following male hairpencil pheromone compounds, which were confirmed to be perceived by female antennae (through electroantennographic detection (EAD) coupled to a GC)^29^: MeSA, δ-decalactone, hexadecanal (16:Ald), hexadecanyl acetate (16:OAc), (*Z*)-7-hexadecenyl acetate (Z7-16:OAc), (*Z*)-11-hexadecenyl acetate (Z11-16:OAc), and hexadecanol (16:OH).

### Larval diet experiment: males reared on full larval diet vs. diet of reduced nutritional value

To determine the effect of larval diet on male hairpencil pheromone composition, we exposed *C. virescens* larvae to two different diet treatments by rearing them on either full (standard) or reduced (25% nutritional value) diets (for details about the diet treatment see^27^). Since the artificial pinto bean diet used for rearing includes plant-based ingredients^31^, we expected that the sequestration of diet-derived compounds would differ between males from the full and the reduced diet. To determine whether males from the full diet (n = 89) had significantly higher amounts of diet-derived compounds in their pheromone than males from the reduced diet (n = 83), we measured the pheromone composition of 2–3 day-old adult males and assessed the differences in mean amounts of the pheromone compounds by performing two-tailed *t*-tests with Bonferroni correction for multiple testing.

### Adult feeding experiment: water fed males vs. sugar-water (10% sucrose) fed males

To test the effect of adult feeding status, i.e., sugar (carbohydrate) consumption, on male hairpencil pheromone composition, we collected 60 adult males on the day of their eclosion and fed half of the group with plain water only (n = 30) and the other half with 10% sucrose solution (n = 30) for two days. On the third day, we extracted the hairpencils of all males during the scotophase. We analyzed the hairpencil extracts by GC following the same procedure as described for the larval diet experiment and compared the average hairpencil pheromone of adult males that were fed water to males fed sugar-water. To determine whether there were significant differences in pheromone composition of water and sugar-fed males, we conducted two-tailed *t*-tests with Bonferroni correction to compare the absolute amounts of MeSA, δ-decalactone, 16:Ald, 16:OAc, Z7-16:OAc, Z11-16:OAc, and 16:OH.

### Two-choice assay to test the relationship between male pupal mass, hairpencil pheromone compounds and female choice

To assess whether MeSA and δ-decalactone content in the male hairpencil pheromone predicted female choice, we included data on MeSA and δ-decalactone content in the hairpencil pheromone of males previously tested in two-choice assays with females (see^27^). In short, in these assays females reared on regular diet were given a choice between two males in an insect cage (30 × 30 × 30 cm; BugDorm, MegaView Science Co., Ltd., Taiwan). The males were reared on either full or reduced diet and fed with 10% sucrose solution as adults. After a mating pair was formed, all individuals were separated. To ensure that mating status would not affect the male pheromone composition, the unchosen male was mated with a virgin female in a separate cup. The following scotophase the hairpencils of both males were extracted. We (re)analyzed the data of 258 males from the study by Zweerus et al.^27^ with a complete record of variables to determine if female choice was related to diet-derived compounds in the male hairpencil pheromone.

To assess whether the diet-derived male pheromone compounds could be indicators of male size, we analyzed the correlation between male pupal mass of the chosen and unchosen males and all their pheromone compounds, including MeSA and δ-decalactone, by calculating Pearson’s correlation coefficients with Bonferroni correction for multiple testing in the software R (version 4.0.5)^33^.

To identify the variables that might affect female choice, we first determined the difference in male pupal mass and the difference in the absolute amounts of the male hairpencil pheromone compounds between the males tested in the same cage. To assess to what extent the difference in male pupal mass (∆ male mass) and the differences in male pheromone compounds predicted female choice, we first randomly selected the data of one male per cage (i.e., this male was either chosen or not chosen by the female). Since each female made one choice in the experiment, this step ensured that the number of data points for the analysis matched the number of choices that females made (n = 129). Subsequently, we modelled the response variable female *choice* as a function of the explanatory variables *∆ male mass* and *∆ each pheromone compound* as additive main effects. We fitted a logistic regression model (glm) and visualized the results using the R package ggplot2^34^. To further investigate how the probability of choice relates to the difference in male pupal mass (∆ male mass) in combination with MeSA or δ-decalactone, we predicted the probability of choice at the mean value of MeSA and δ-decalactone related to three different levels (i.e., mean (± sd)) of ∆ male mass (marginal effects). We conducted the analyses in the software R (version 4.0.5)^33^ and visualized the model output using the packages ggeffects^35^, sjPlot^36^ and margins^37^.

## Results

### Effect of larval diet and adult feeding on male hairpencil pheromone

**Figure 1:**
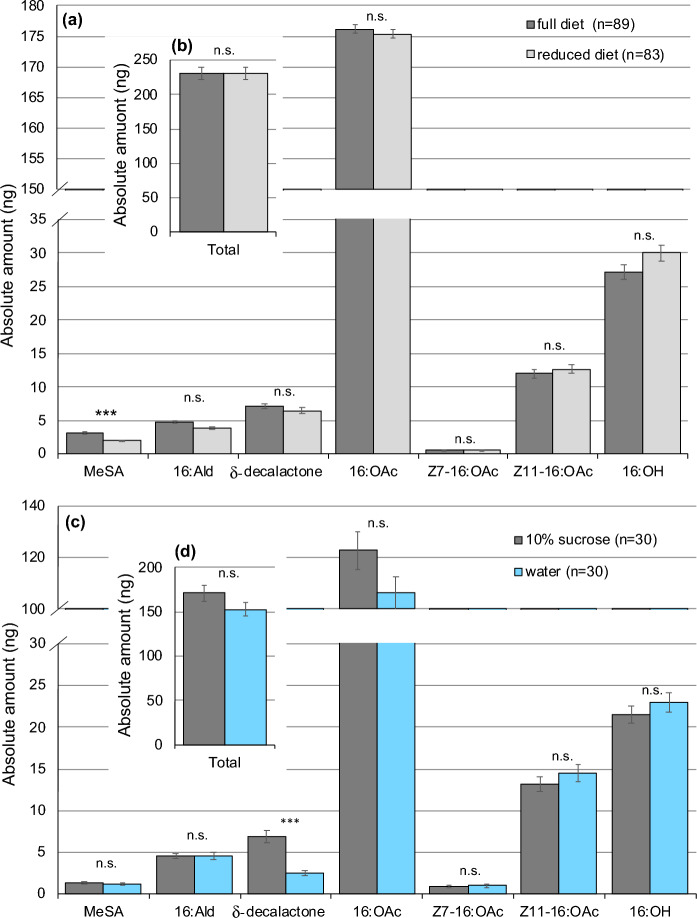
Effect of larval diet (**a**, **b**) and adult feeding (**c**, **d**) on male hairpencil pheromone. (**a**) Average absolute amounts of male hairpencil compounds and (**b**) average total amount of hairpencil pheromone of males from full diet (dark grey bars) vs. reduced larval diet (light grey bars) that were fed sugar water as adults. (**c**) Average absolute amounts and (**d**) average total amount of hairpencil pheromone of males that were reared on full larval diet and then fed with water (blue bars) or sugar-water (grey bars) as adults. Error bars: ± se. *** *P* ≤ 0.001, n.s., not significant.

Hairpencil extracts of males reared on a regular diet as larvae contained a significantly higher absolute amount of MeSA than males reared on reduced diet (two-tailed *t*-test, *t* = 5.161, df = 170, *P* ≤ 0.001, Fig. 1a). The two diet treatments had no effect on the absolute amount of any other pheromone compounds (all *P* ≥ 0.05, Fig. 1a), including the major compound 16:OAc (*t* = 0.070, df = 170, *P* = 0.944, Fig. 1b).

When males were reared on regular diet as larvae and given sugar water as adults, their hairpencils contained significantly higher amounts of δ-decalactone than males that were given water only as adults (two-tailed *t*-test, *t* = 5.769, df = 58, *P* ≤ 0.001, Fig. 1c). The absolute amount of all other pheromone compounds and the total amount of male hairpencil pheromone were not significantly different between water-fed and sugar-fed males (all *P* ≥ 0.05, Fig. 1d).

### Relation of male pupal mass and hairpencil pheromone compounds and mating success

**Figure 2:**
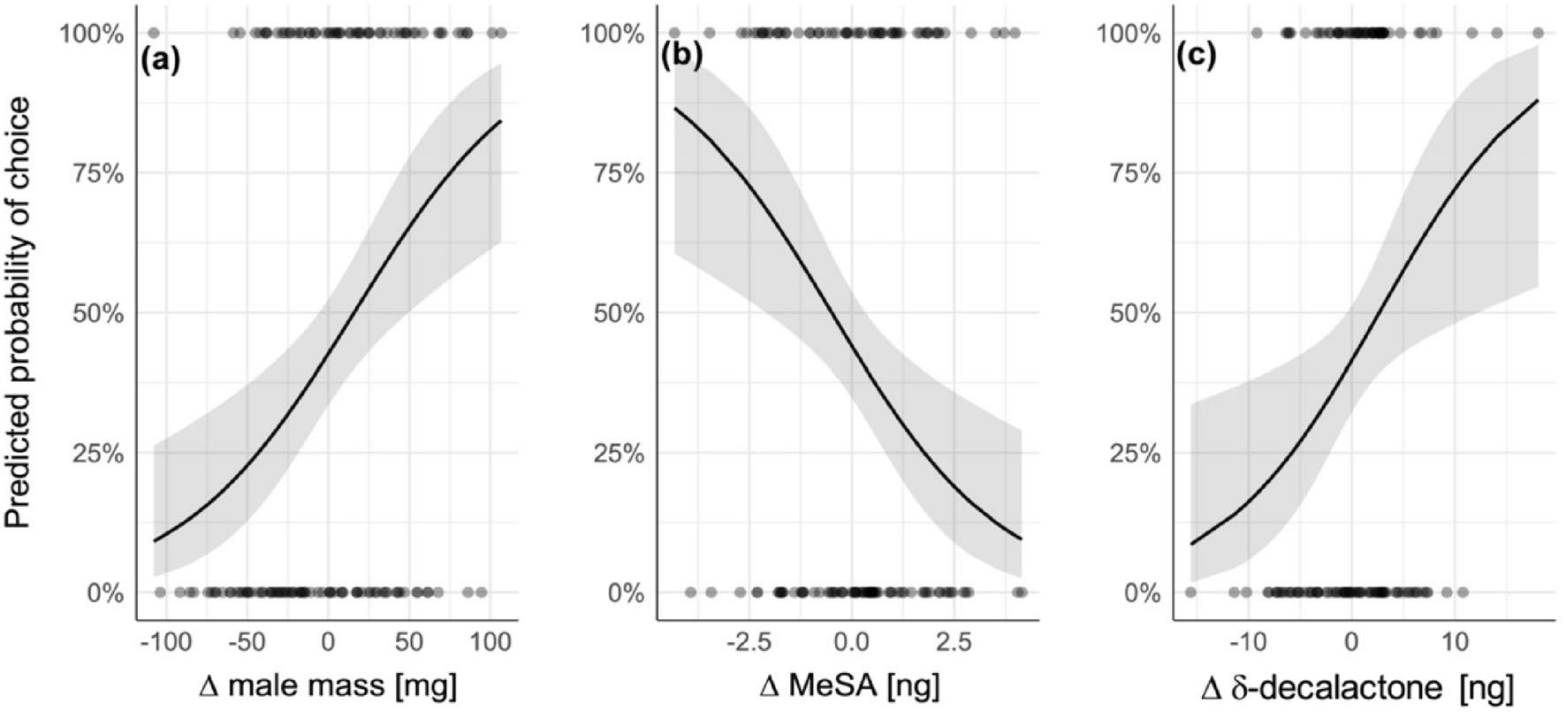
Results of two-choice tests with females. Marginal effects, i.e., the predicted male’s mating probability as a function of (**a**) the difference in male pupal mass, (**b**) the difference in the amount of MeSA in the hairpencils and (**c**) the difference in the amount of δ-decalactone in the hairpencils while all other independent variables were held constant. The curve (solid line) shows the predicted male mating probability. Shaded area indicates 95% CI. Dots mark actual data points.

We used logistic regression to predict a male’s mating pobability as a function of the difference in mass between the two males in the cage and the difference in the pheromone compounds of these males. The differences in male pupal mass significantly affected male mating probability (Fig. 2a), with heavier males being more likely to mate (Z = 3.385, *P* = 0.001, Table 1). We found that both MeSA (r_p_ = 0.45, *P* = 0.008) and δ-decalactone (r_p_ = 0.30, *P* = 0.008) correlated positively with male pupal mass and that this correlation was stronger for these hairpencil compounds than for any other compounds (Fig. S1, Supplementary material). Surprisingly, males that produced more MeSA than their competitor were less likely to mate than the males that produced less MeSA (Z = − 3.010, *P* = 0.003, Fig. 2b). Conversely, males that produced more δ-decalactone than their competitor were more likely to mate (Z = 2.507, *P* = 0.012, Fig. 2c). None of the other hairpencil pheromone compounds affected female choice (all *P* > 0.05, Table 1).

**Table 1:** Analysis of Deviance table for the logistic regression analysis of the two-choice tests.

Variable	ß	SE	Z	*P*-value	e^ß^	95% CI for e^ß^
**∆ male mass**	0.019	0.005	3.385	**0.001**	1.019	[1.008, 1.030]
**∆ MeSA**	− 0.486	0.161	− 3.010	**0.003**	0.615	[0.440, 0.832]
∆ 16:Ald	− 0.112	0.081	− 1.392	0.164	0.894	[0.760, 1.045]
**∆ δ-decalactone**	0.130	0.052	2.507	**0.012**	1.138	[1.033, 1.267]
∆ 16:OAc	− 0.002	0.003	− 0.501	0.616	0.998	[0.992, 1.005]
∆ Z7-16:OAc	0.400	0.674	0.558	0.557	1.487	[0.400, 5.781]
∆ Z11-16:OAc	0.040	0.032	1.224	0.221	1.040	[0.977, 1.110]
∆ 16:OH	− 0.003	0.017	− 0.161	0.872	0.997	[0.965, 1.031]

Significant variables are listed in bold.

ß = estimated regression coefficient, i.e., the expected change in log odds that a male was mated per unit change of the variable, with SE = standard error and Z = test statistic for the regression coefficient. e^ß^ is the odds ratio that a male mates for every unit increase of the variable with 1 (e.g., if the mass difference between two males increases by 1 mg, then the odds that the larger male is mated increases 1.9%).

## Discussion

We investigated if two newly described male hairpencil pheromone compounds could be involved in female mate choice in the noctuid moth *C. virescens*. Methyl salicylate (MeSA) and δ-decalactone are both diet-derived and at least MeSA is sequestered from the larval and the adult diet^29^. We found that well-fed males that were reared as larvae on regular diet produced more MeSA but not δ-decalactone in their pheromone than males reared on a reduced diet. Sugar-fed adult males contained higher levels of δ-decalactone but not MeSA in their hairpencil pheromone compared to males fed plain water. The amounts of both compounds were positively correlated to male pupal mass. Since male pupal mass is a measure of male quality in *C. virescens*^27^, we hypothesized that females may use these compounds as measures of male quality. In reanalyzing female choice data, now including the newly discovered compounds MeSA and δ-decalactone, we found that the probability that a female chooses a male was negatively affected by the amount of MeSA the male produced and positively affected by male mass and the amount of δ-decalactone in the male hairpencil pheromone. While little is known about the biosynthesis and degradation of δ-decalactone and MeSA, and the mechanism by which females choose males based on δ-decalactone and/or MeSA remains unknown, both compounds are perceived by females^29^ and have the potential to inform females about male size and hence, quality.

### Previously identified hairpencil compounds do not affect female choice

We confirmed that none of the previously identified hairpencil compounds (16:Ald, 16:OAc, Z7-16:OAc, Z11-16:OAc, and 16:OH)^26^ explained female choice. These compounds are biosynthetically related to compounds found in the female sex pheromone. However, the newly discovered compounds MeSA and δ-decalactone both affected female choice, even though MeSA in a negative and δ-decalactone in a positive way. This suggests that females might assess males based on these two chemical signals that males emit from their hairpencils during courtship. Since these compounds are biosynthetically different from and not present in the female sex pheromone, selection pressures affecting the male and female pheromone are likely different.

### MeSA in hairpencils is not attractive to females

Liu et al.^29^ found that males sequester MeSA as larvae and that the mating rate of hairpencil-ablated males that were supplied with different amounts of MeSA (0–100 ng) increased with increasing amounts of MeSA. Surprisingly, we found a negative relation between the amount of MeSA that males produced and their mating probability (Fig. 2). The apparent difference in the results of Liu et al.^29^ and our results on the effect of MeSA on a male’s mating probability might be explained in several ways. First, in the experiments conducted by Liu et al.^29^, the mating rate was measured in no-choice assays, using one male and one female in a mating cup. The mating rate of males with ablated hairpencils was similar to the mating rate of unablated control males if the ablated males were provided with sufficient MeSA^29^. In our experiments, we offered each female a choice of two intact males. An alternative explanation for the negative correlation between a male’s MeSA content and female preference is that MeSA is used as an anti-aphrodisiac. In the green-veined butterfly *Pieris napi,* males produce MeSA from L-phenylalanine acquired during larval and adult feeding and the males provide MeSA to females in their spermatophore, making these females less attractive to other males^38–41^. Using their hairpencils, male *C. virescens* also provide anti-aphrodisiac pheromone (16:OAc) to females during copulation^22^. Possibly, MeSA is an additional anti-aphrodisiac component of the male pheromone that polyandrous females prefer to avoid when given a choice between two males.

### Delta-decalactone may signal male quality

The amount of δ-decalactone in *C. virescens* hairpencils was positively correlated with male pupal mass (Table 1). Since male pupal mass is a measure of male quality and translates into male size^27^ and females preferred males with a higher δ-decalactone content (this study), females might use δ-decalactone to gauge male quality. To our knowledge, δ-decalactone is rare in lepidopterans. It has been reported as a male-specific compound in *Heliconius* butterflies^42^ and in the lycaenid butterfly *Celastrina argiolus*, which contain δ-decalactone in specialized scales on the male wings^43^. It is unknown if δ-decalactone is synthesized de novo in lycaenid butterflies and if it also plays a role in female choice in this butterfly. Delta-decalactone can be synthesized from linoleic acid^44^. Since Lepidoptera cannot biosynthesize polyunsaturated acids, such as linoleic acid, this precursor likely comes from the diet^45–47^. Surprisingly, we found that sucrose feeding increased the amount of δ-decalactone in the hairpencils of C. *virescens* males*.* We do not know if *C. virescens* males are able to synthesize δ-decalactone only if sufficient carbohydrates are available, or if the males transfer more sequestered δ-decalactone to the hairpencils when they are well-fed. Since adult males can adjust the δ-decalactone content in their hairpencils through sugar feeding, it seems less likely that this compound only signals male size to the females. Possibly, larger, higher quality males sequestered more δ-decalactone precursor in the larval stage and/or more easily achieve a well-fed status by feeding on nectar to produce or sequester sufficient δ-decalactone. A male’s δ-decalactone content in the hairpencils might thus signal that a male is healthy and well-fed.

### Relative mate choice can only be revealed in two-choice assays

Previously, we found that mating latency of antennectomized females and intact females did not differ significantly^27^. If female mating latency is a reliable measure of female choice, it would appear that female choosiness is unaffected by pheromone perceived via the female’s antennae^27^. However, Lepidoptera also have olfactory receptors on their abdomen and legs^48,49^. Females may therefore still assess males based on MeSA and δ-decalactone. Notably, the experimental designs of the mating latency experiment and the female choice assay differed significantly. Zweerus et al.^27^ determined mating latency in no-choice assays while female choice in this study was assessed in two-choice assays. No-choice assays can be informative by comparing metrics of courtship and mating across treatments, as reported by Zweerus et al.^27^ and Liu et al.^29^ for mating latency and cumulative mating over time, respectively. However, because *C. virescens* females choose males based on relative criteria, two-choice assays are essential to investigate female choice.

### Further insight into female choice could be gained by manipulating males

Other experimental approaches, such as testing synthetic compounds in augmentation experiments, may seem obvious alternatives to measure female choice in *C. virescens*. However, in our many attempts to perfume males with MeSA and δ-decalactone, we encountered several drawbacks. First, to minimize the effects of variation in endogenous compounds, the hairs (scales) of the male hairpencils need to be surgically ablated (see^16^), which introduces significant injury to the males. This procedure also leaves behind some pheromone components (e.g.,^29^) and the cells that secrete the pheromone. Second, applying the compounds to the hairpencils requires manual eversion of the hairpencils or injection of the compounds in a solvent into the “pouch” (see^29^), which also bear a high risk of injuring the male. With both approaches, it is challenging to design appropriate sham controls, making it difficult to distinguish the effects of injury from the subtle effects of synthetic blends on the relative attractiveness of males. Therefore, we found augmentation experiments unsuitable to answer the questions raised in this study. An exciting approach to test the effects of synthetic blends of compounds on female choice in *C. virescens* and other moths would be to knock out pheromone-producing genes or transport molecules that deliver the pheromones to the tissues that emit them. However, this strategy will need to await the identification of the genes involved in these processes.

### Sensory biases shape pheromone evolution

In conclusion, we found that two newly identified diet-derived compounds in the male hairpencils are likely part of the male sexual signal that is used in female choice. Since females generally attract multiple males by emitting a long-range sex pheromone, such choices likely occur in nature^50^. MeSA and δ-decalactone are both plant compounds that are abundant in the environment of the females; MeSA is a major herbivore- and pathogen-induced plant volatile^51–53^ and decalactone is associated with sugar-rich foods, such as fruits and flowers^54,55^. Receptors that are tuned to these odours^29^ and have evolved under natural selection may thus have been exploited by males to attract the attention of females during courtship, an example of “sensory bias”^56^. Male hairpencil volatiles thus function as both interspecific cues and intraspecific sexual signals, which likely make them subject to both directional and stabilizing selection.

### Supplementary Information

Supplementary Figure S1.

## Data Availability

The datasets analysed for the study are available from the corresponding author on request.
