# Supplementary material for: Diet-derived male sex pheromone compounds affect female choice in a noctuid moth
Source: Sci Rep. 2023 Nov 13;13:19802. doi: 10.1038/s41598-023-47041-8 (PMC10643667; doi:10.1038/s41598-023-47041-8)
Supplement: Supplementary file 1 — Supplementary Figure S1. [file 41598_2023_47041_MOESM1_ESM.pdf]

Manuscript: Diet-derived male sex pheromone compounds affect female choice in a noctuid moth

Supplementary material

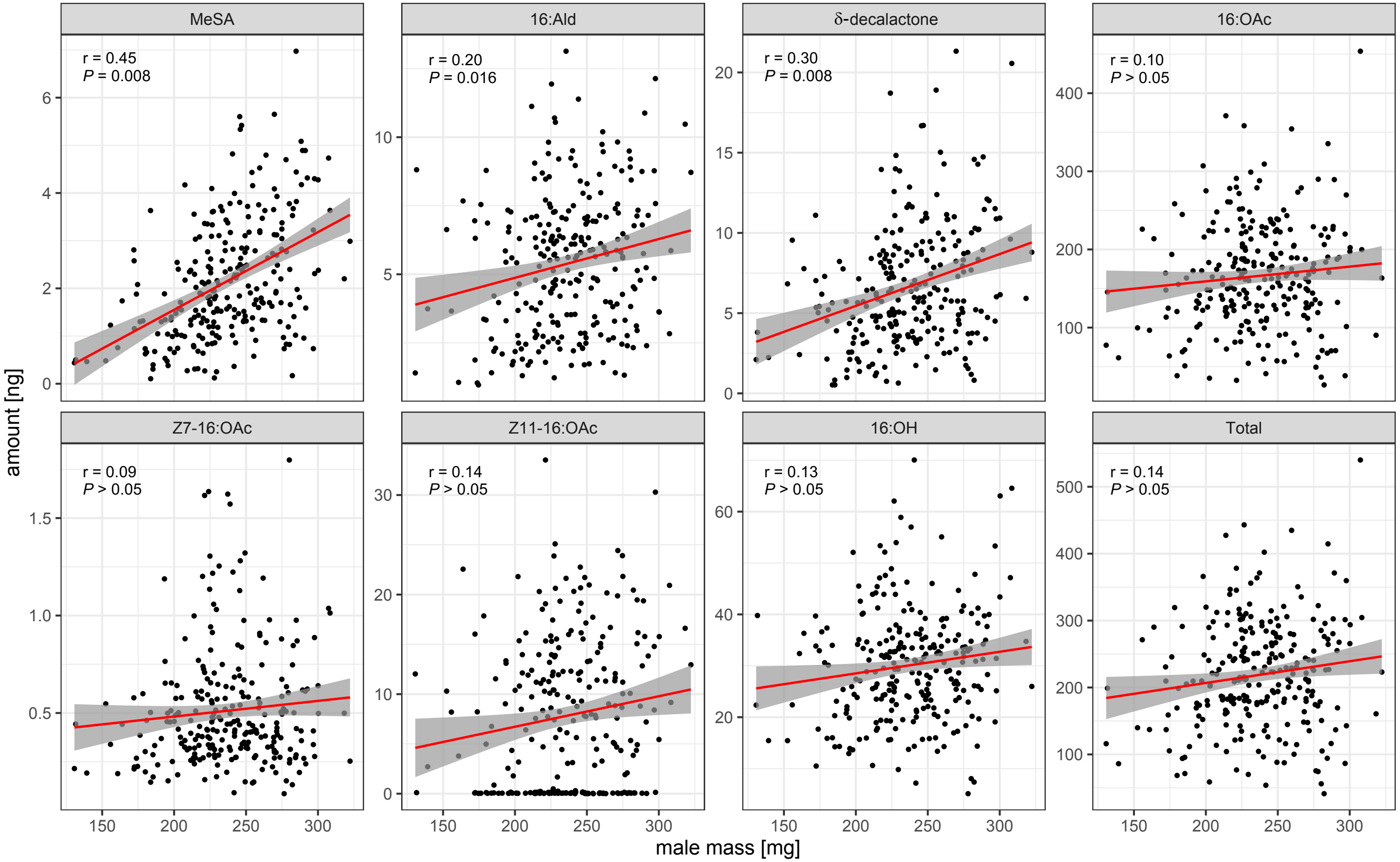

Fig. S1 Scatter plots showing correlations between male pupal mass and absolute amounts of male hairpencil pheromone compounds. The correlation coefficient r (Pearson's r) and *P*-values are reported in the top left of each panel.
